# Efficient, high-power, narrow-linewidth, continuous-wave quantum-dot semiconductor comb laser

**DOI:** 10.1038/s41598-024-53609-9

**Published:** 2024-02-20

**Authors:** Mikhail Buyalo, Alexey Gubenko, Sergey Mikhrin, Vladimir Mikhrin, Alexey R. Kovsh, Ashok V. Krishnamoorthy

**Affiliations:** 1 Innolume GmbH, Dortmund, Germany; 2 Alfalume Inc., Los Gatos, CA USA; 3 Axalume Inc., San Diego, CA USA

**Keywords:** Mode-locked lasers, Semiconductor lasers, Diode lasers

## Abstract

We report a continuous-wave, O-band quantum-dot semiconductor comb laser for WDM optical interconnects exhibiting a 2.2 THz optical bandwidth with up to 89 comb wavelengths spaced at 25 GHz, over 30% peak ex-facet electrical-to-optical power conversion efficiency, up to 270 mW of usable laser power, relative intensity noise below − 135 dB/Hz per individual mode, individual laser mode linewidth of 140 kHz, mode beating linewidths of 50 kHz across all modes, and stable far-field output with 75% coupling efficiency to PM fiber in a butterfly package.

## Introduction

The unabated growth in datacenter compute and switching bandwidth has driven an ever-increasing demand for reliable, energy-efficient and cost-effective optical interconnect. A number of low-voltage, high-speed modulator array technologies, including silicon microring modulators^1–5^ and lithium niobate on insulator modulators^6^, have been developed to meet this challenge. Resonant silicon microring modulators, when cascaded on a waveguide bus, have demonstrated the ability for dense WDM links with bandwidth and energy-efficiency in the low picojoules/bit^7–11^. But an energy-efficient, reliable, high-power, scalable-wavelength comb source has proved elusive.

Inspired by the promise of three-dimensional confinement of carriers^12^, early work on room temperature quantum dot lasers showed potential for very low threshold currents^13–15^, O-band operation^16^, 45% power conversion efficiency (PCE) and up to 3.5 W of continuous-wave (CW) power^17^. Fabry–Perot lasers based on self-assembled InAs quantum dots grown on GaAs substrates demonstrated early potential for lasing over as many as 1000 wavelengths across 14 THz^18^. Driven by applications in sensing, metrology, and optical communications, we have witnessed renewed interest in integrated, pulsed semiconductor mode-locked lasers^19,20^. Passively mode-locked quantum-dot comb lasers^21,22^ together with silicon microring modulators have been investigated for optical interconnects with promising results^23–26^. However, a continuing challenge for comb lasers for short-distance communication and computing applications has been to concurrently achieve adequate power per line, uniform output power over a wide spectral range, low noise per line, narrow linewidth, and notably high energy efficiency—a key requirement for optics in computing systems^27^.

**Figure 1:**
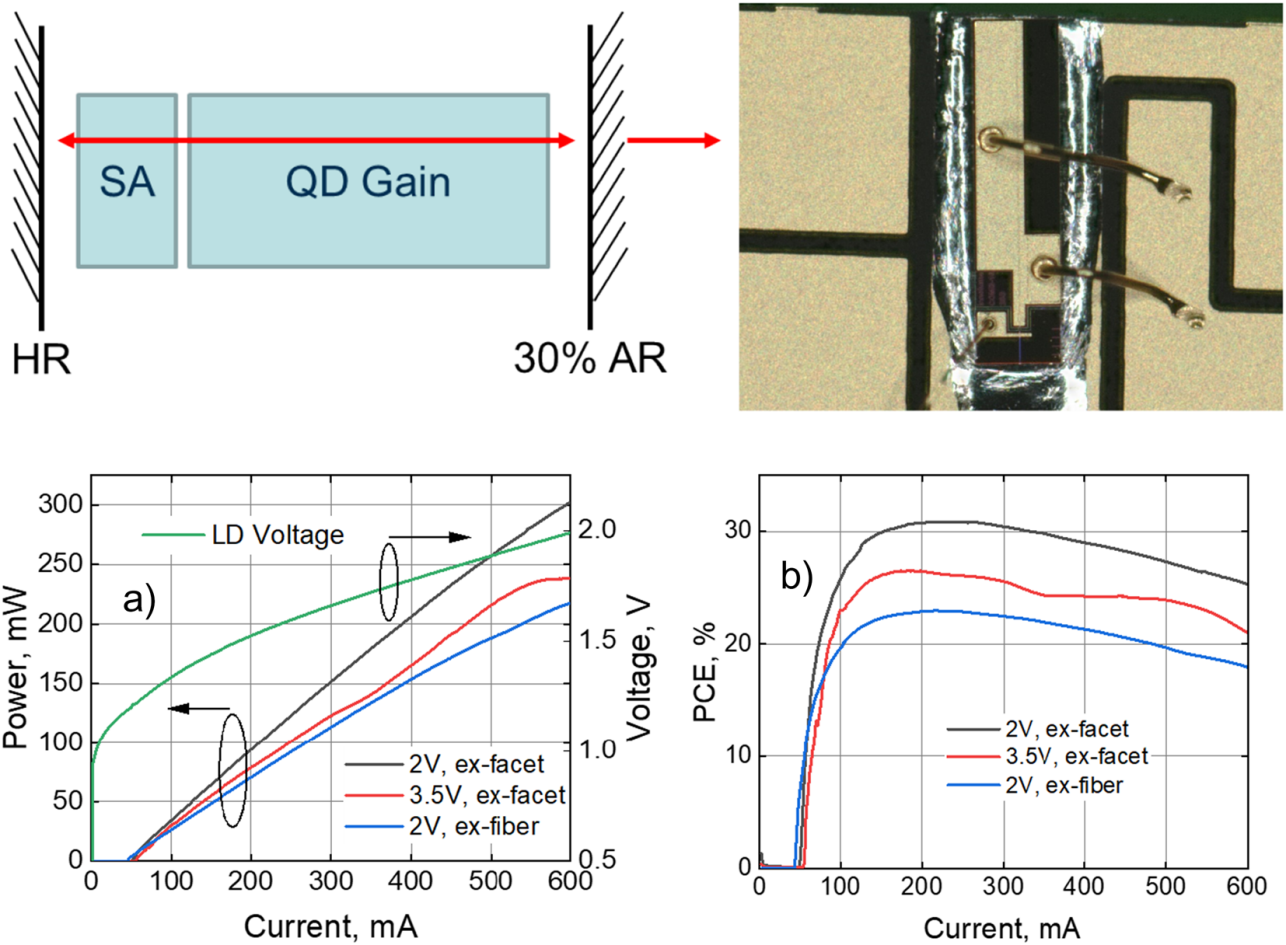
25G10-layer quantum dot comb: (**a**) laser schematic; (**b**) photograph of the laser die-bonded and wire-bonded to a ceramic carrier; (**c**) L–I–V; and (**d**) PCE characteristics at different absorber bias values, measured ex-facet and ex-fiber in a butterfly package.

Here we show that passively mode-locked quantum-dot comb lasers with wavelength spacings in the range of 25–100 GHz can be operated the individual modes being phase correlated and exhibit excellent output power, efficiency, uniformity and spectral properties. At 25 GHz wavelength spacing, the O-band comb lasers achieve stable, CW performance across an operating bandwidth over 2 THz, Lorentzian linewidths of 140–170 kHz, peak PCE over 30% ex-facet at 25 °C at 200 mA drive current, and ex-facet power above 250 mW at 500 mA drive current (Fig. 1). Stable far-field output angles of 5°–7° and 27° in the slow- and fast-axes respectively, enable polarization-maintaining fiber-coupling efficiencies of approximately 75% of peak power after assembly into butterfly packages. When used with a 1xN cyclic demux or wavelength de-interleaver to create a parallel multi-wavelength transmitter with Nx25 GHz spacing, it becomes possible to use a single comb source and extend the bandwidth per wavelength to be side-band crosstalk-limited by the de-interleaved wavelength spacing of the comb for non-return-to-zero or multi-level amplitude modulation^28,29^, or OSNR-limited for coherent communication^30^.

### Comb laser performance

The structure of the comb laser is based on a simple two-section device with a gain and absorber section. The cleaved Fabry–Perot laser is high-reflectivity coated near the absorber end and 30%-reflection coated at the opposite end to achieve a single-sided output laser. The epitaxial structure was optimized individually for each of the lasers, taking into account that the shorter cavities require higher modal gain to overcome radiative losses. We report three comb lasers with the following characteristics: a 100 GHz comb with twelve quantum dot layers and a cavity length of 400 um; a 50 GHz comb with ten quantum dot layers and a cavity length of 830 μm; and a 25 GHz comb with ten quantum dot layers and a cavity length of 1600 μm. In each case, the lasers have ridge waveguides with a mesa width of 3 μm, and the absorber length is approximately 10% of the corresponding cavity length. As a result, our 100 GHz spacing comb has largest amount of QD layers and highest optical confinement with a vertical (fast-axis) full-width half-max (FWHM) beam divergence of 67° and a horizontal (slow-axis) beam divergence of 35° FWHM. The FMHW beam divergences of the 50 GHz comb lasers are 40° and 10° in the corresponding fast and slow axes, whereas in the 25 GHz comb, 27° and 6° respectively. Measurements were taken at peak operating current across a batch of lasers fabricated on the same wafer, with a standard deviation of 1.5° for the 100 GHz comb, 1.1° for the 50 GHz comb, and 0.4° for the 25 GHz comb on both axes. Figure 1 shows the forward light–current-voltage characteristics of the two-section quantum dot laser diode taken with its absorber section reverse biased at 2 V and 3.5 V, corresponding to comb regimes of high-efficiency/high-power and large wavelength span, respectively. Power levels are measured at both chip output facet and PM fiber output after coupling and packaging into a 14-pin butterfly package. As reverse bias is increased, PCE (defined as optical power out/electrical power in) degrades due to the increased photocurrent in the absorber, and there is evidence of nonlinearity in the LI curve due to shifts of the entire mode spectrum as current drive changes. An ex-facet PCE of 30% is seen in the high-efficiency regime (150–300 mA) with 2 V absorber reverse bias, reducing to 25% at −2 V/600 mA (high-power) and to 24% at −3.5 V/500 mA (wide-bandwidth). For an identical fiber-coupled laser with its absorber reverse-biased at 2 V, an ex-fiber efficiency above 20% is maintained in the operating current range of 110 mA to 480 mA.

**Figure 2:**
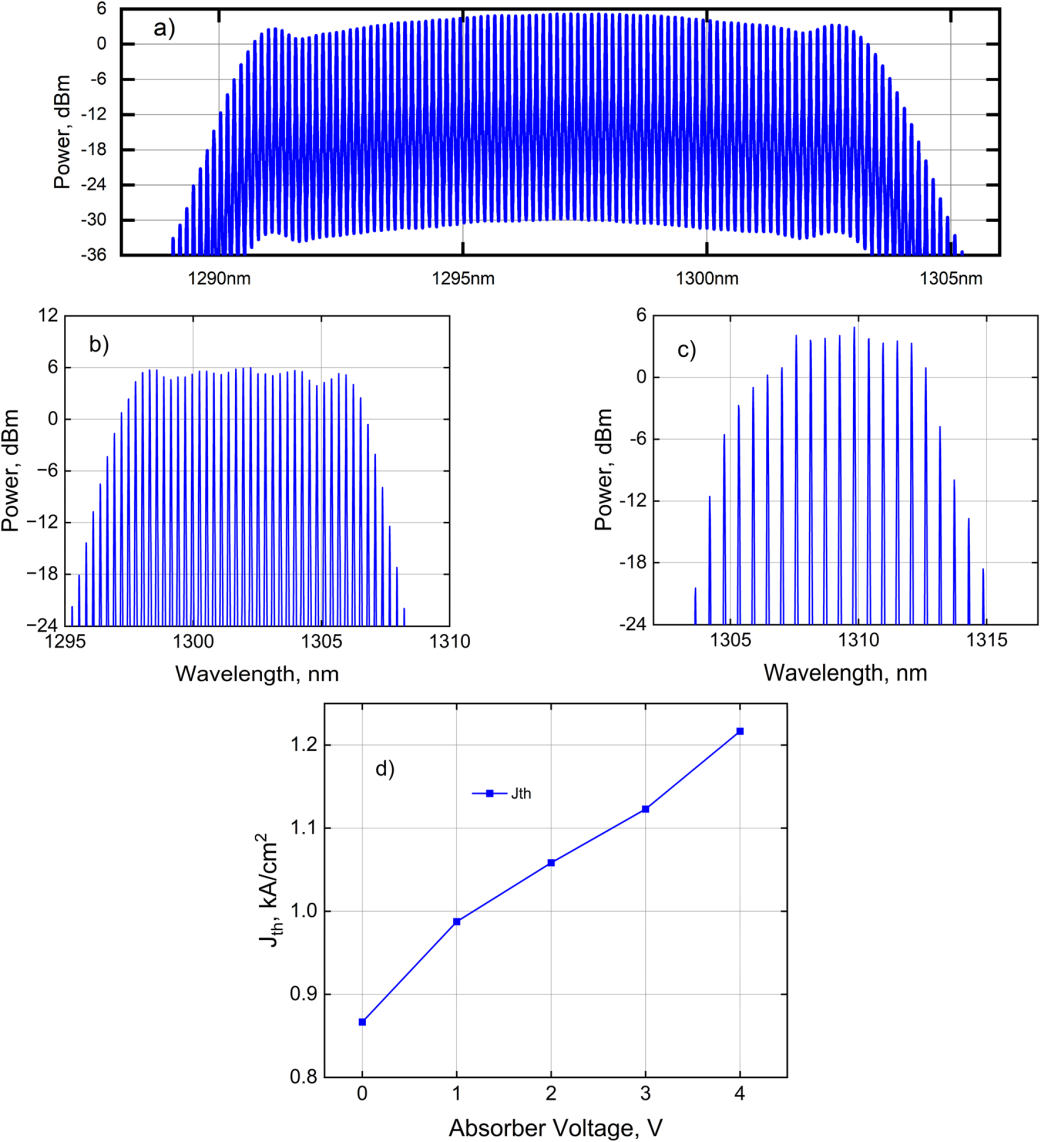
Optical spectrum of: (**a**) a 1.6 mm 25G comb laser at 500 mA with 84 consecutive modes ≥ 1.5 mW at −3.5 V absorber bias; (**b**) a 0.83 mm 50G comb at 300 mA with 31 consecutive modes ≥ 2 mW (18% PCE) at −5 V absorber bias; (**c**) a 0.4 mm 100G comb at 85 mA with 12 consecutive modes ≥ 1 mW (15% PCE) at 0 V absorber bias; (**d**) threshold current density of the 25G comb laser versus absorber reverse-bias voltage. Absorber lengths are 10% of respective cavity lengths.

The measured CW spectrum of the comb laser with 25 GHz spacing at a drive current of 500 mA and absorber reverse bias of 3.5 V is shown in Fig. 2a. 89 modes are observed with a power of ≥ 1 mW each. Within a ± 2 dB range, 84 consecutive cavity modes are available with an average power of 2.5 mW/wavelength providing a usable power of 210 mW (out of a total laser output power of 216 mW). If the usable range is narrowed to ± 1.5 dB, the number of modes drops to 74, with an average power of 2.6 mW/wavelength for a usable power of 192 mW. The corresponding spectrum for a comb laser with 50 GHz spacing driven at 300 mA and 5 V reverse bias is shown in Fig. 2b. Here, we see 31 consecutive modes spaced at 50 GHz with a power over 2 mW (3 dBm) per mode or equivalently 31 consecutive modes within a 3 dB range with an average power of 3.5 mW/mode. Figure 2c shows a comb with 100 GHz wavelength spacing measured at a drive current of 85 mA with 12 consecutive modes above 1 mW/mode, whereas within a 3 dB range, there are 9 modes with an average power of 2.7 mW/mode. All spectra were measured with the lasers biased well below their maximum (rollover) currents. The threshold of the comb laser was investigated as a function of reverse bias. As shown in Fig. 2d, this value varies between 0.8 and 1.2 kA/cm^2^ given the mesa width of 3 microns and length of 1.6 mm neglecting current spread below the mesa.

**Figure 3:**
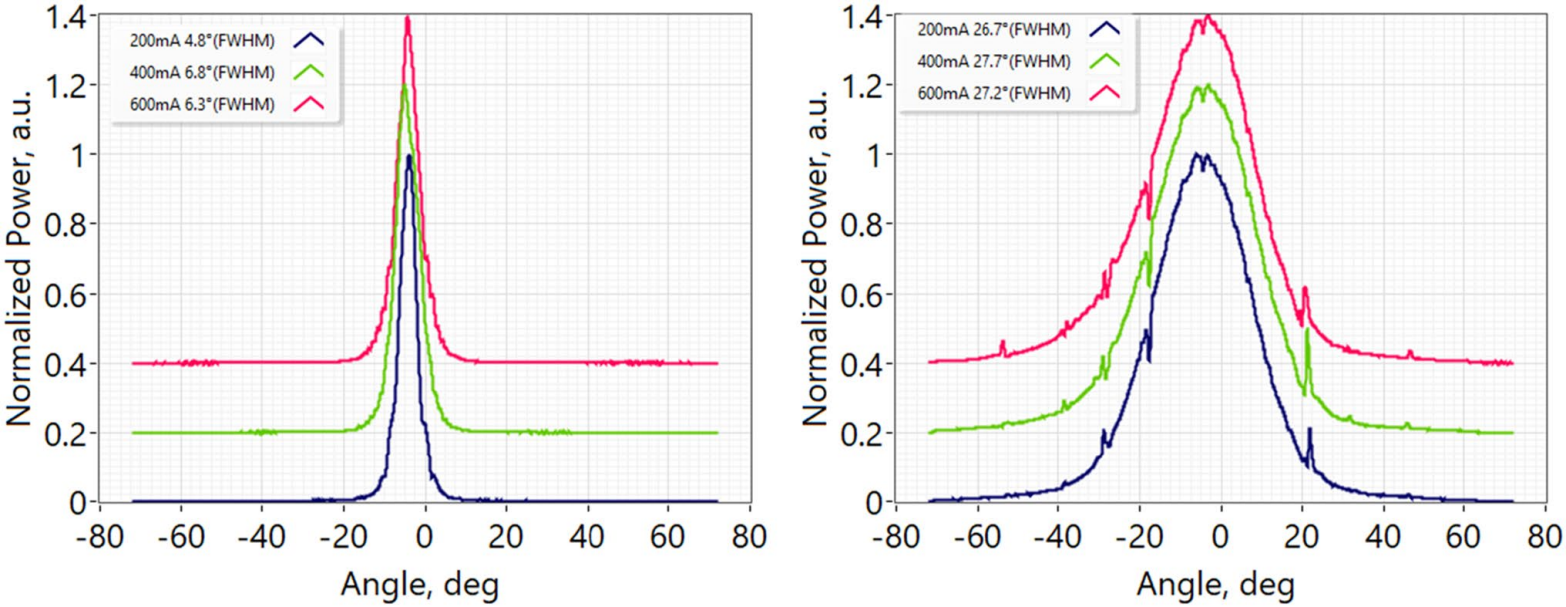
Far-fields of the 25G-spacing comb laser for various drive currents showing horizontal in-plane (left) and vertical full-width, half-maximum divergence angles.

**Figure 4:**
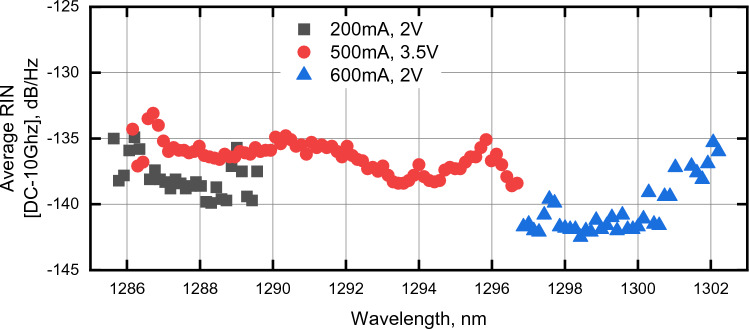
Averaged RIN of individual 25G COMB modes across DC to 10 GHz vs laser drive current and absorber reverse bias.

The far-field of the 25G comb laser was characterized as a function of drive current. As shown in Fig. 3, far-field output angles in the range of 4.8°–6.8° and 26.7°–27.2° were measured for the slow- and fast-axes respectively. A second sample of the 25G comb laser was assembled into a 14-pin butterfly package and the average RIN across 10 GHz for each filtered mode was measured for various drive currents and absorber reverse bias. Figure 4 plots the RIN results for each filtered mode when the comb was biased for peak efficiency (200 mA, −2 V, 32 modes, 87 mW usable power ex-facet), widest bandwidth (500 mA, −3.5 V, 76 modes, 213 mW usable power ex-facet), and highest power (600 mA, 2 V, 39 modes, 270 mW usable power ex-facet), respectively. The RIN (averaged to 10 GHz) per mode was below −135 dB/Hz in the wide-bandwidth regime and below −140 dB/Hz in the high-power regime (Fig. 4).

**Figure 5:**
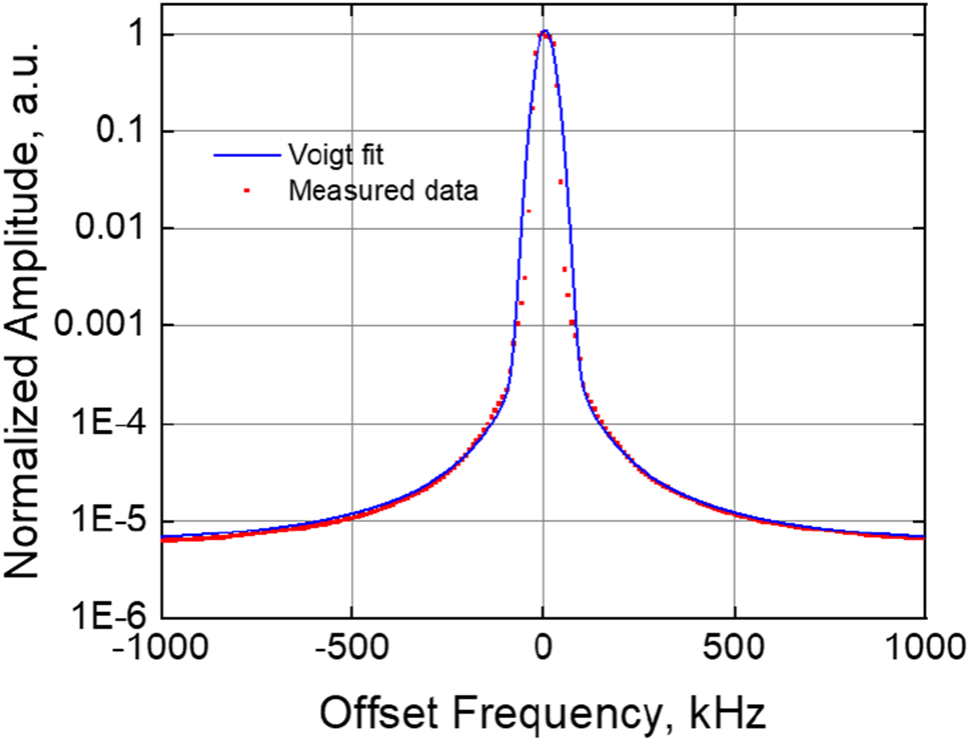
Beating linewidth of entire 25G COMB at 200 mA current, 2 V absorber reverse bias, and acquisition time 60 s (dots show measured data, solid lines represent corresponding Voigt fits).

Above 100 mA, the laser operates without pulses which can be attributed to the broadening of the mode-locked pulses beyond the pulse repetition interval associated with the cavity length of the comb laser^31^. The pulse broadening relates to the chromatic dispersion of the effective refractive index that results in nearly quadratic phase separation between modes^32^. In this regime, stable, continuous-wave multi-wavelength comb spectra are observed. Further increasing the reverse bias voltage blue-shifts the comb and reduces output power and efficiency. Transition to frequency modulation in mode-locked quantum dot comb lasers based on the same material system at high drive currents was recently reported^33^. The 25G comb achieves a maximum span of 12 nm (2.14THz) at −3.5 V absorber bias demonstrating 89 consecutive modes with over 1mW/mode (Fig. 1a). In order to measure mode-spacing stability, the output of the packaged 25G comb, driven at 200 mA and 2 V reverse absorber bias (peak efficiency regime), was filtered through a linear dispersive medium (chirped fiber Bragg grating) to convert frequency modulation to amplitude modulated pulses. The down-converted RF spectrum of the pulses, shown in Fig. 5, represents the combined mode-beating linewidth of all the lasing modes of the comb. The mode spacing of the comb was 25.56 GHz and the mode beating linewidth (FWHM) of all lasing modes acquired over 60 s was 49.5 kHz or equivalently a comb frequency stability of 2 ppm.

**Figure 6:**
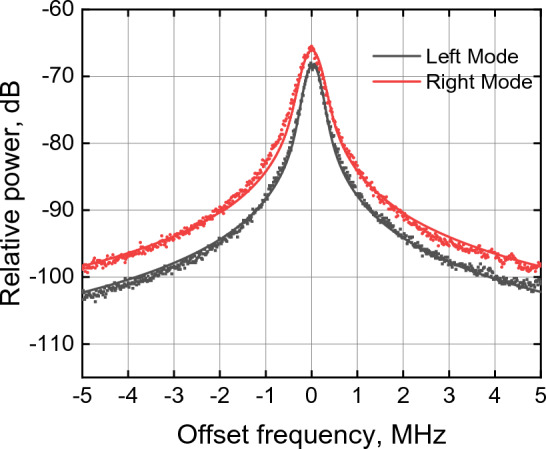
Linewidth measurement of two different free-running 25G COMB modes at 28 °C (without thermal stabilization); gain and absorber section are driven from batteries with 200 mA and 1.5 V, respectively; dots show measured data, solid lines represent corresponding Voigt fits.

The linewidths of the packaged, uncooled 25G comb were measured in the peak PCE operating regime at 210 mA current (approximately 4 × threshold current) and 1.5 V reverse absorber bias using a self-heterodyne method. Benefitting from the low linewidth broadening (α_H_) factor associated with the multi-layer quantum dot Fabry–Perot lasers^34^, low linewidths may be expected from individual modes. Two modes of the comb spaced 2 nm apart were measured in the central region of the comb near the gain peak. As shown in Fig. 6, the beating line data is closely fit to a Voigt profile. The extracted Gaussian component of the optical linewidth (related to 1/f noise) is 316 kHz and 367 kHz FWHM for two respective modes. The natural Lorentzian optical linewidth (related to white frequency noise) is 140 kHz and 170 kHz respectfully (see “Methods”: “Comb linewidth measurement and analysis”). Since the modes are locked to each other, one can expect the same width of each line within the mode-locked spectrum. The slightly increased linewidth for the longer lasing wavelength may be attributed to the dispersion of the α_H_ factor across the lasing spectrum^35^.

In summary, we present CW, O-band quantum-dot semiconductor comb lasers with spacings of 100, 50, and 25 GHz, exhibiting an optical bandwidth of 2.2 Thz (12 nm) with up to 89 comb wavelengths spaced at 25 GHz, record 30% peak ex-facet PCE, 270 mW of usable laser power, comb linewidths of 140 kHz, and average RIN below −140 dB/Hz per mode, making them compelling sources for parallel WDM optical interconnects. These combs may be directly used as a multiwavelength source or interfaced to an N-channel de-interleaver to create N comb sources with Nx25 GHz wavelength spacing in separate waveguides with 84/N wavelengths per waveguide.

## Methods

### Comb laser butterfly assembly

25 GHz spaced comb lasers were assembled into two types of 14-pin butterfly packages. In the first type, the laser output was guided with a combination of fast and slow axis collimators through double stage isolator into a polarization maintaining (PM) single mode fiber with a collimator on its end. This assembly ensured absence of parasitic reflections to the comb laser, allowing ease of study of its RIN and linewidth performance. In the second type of 14-pin butterfly the comb emission was collected by a fiber with an anti-reflection-coated cylindric lens on the fiber end, enabling high coupling efficiency. Thermal chip stabilization was ensured by a thermistor, bonded on the chip carrier adjacent to the laser diode and Peltier cooler.

### Line spacing stability measurements

**Figure 7:**
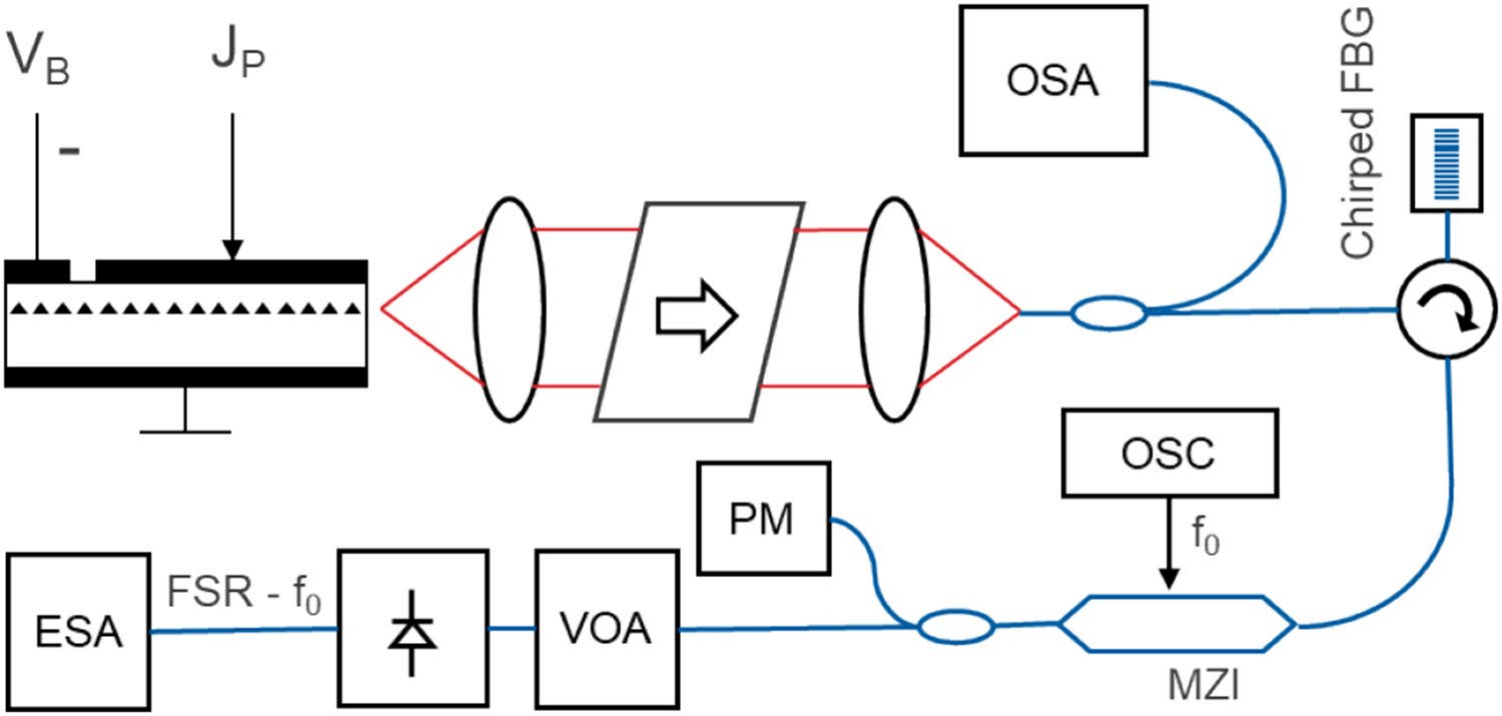
Setup for comb line-beating experiment showing clockwise from top left the FP comb laser with drive and absorber bias sections, a free space isolator and fiber coupler (in a laser butterfly package), a 10/90 splitter with an optical spectrum analyzer (OSA) and a circulator with a chirped fiber bragg grating reflector, a high-speed MZI (24 GHz) driven by a signal oscillator (OSC) for down-conversion of the comb beat frequency, a 10/90 splitter with a power monitor (PM) and variable optical attenuator (VOA) followed by a high-speed photodetector and an electrical spectrum analyzer (ESA). Polarization-maintaining (PM) fibers were used throughout (blue lines).

The output of the packaged 25G comb, driven at 200 mA and 2 V reverse absorber bias (peak efficiency regime), was isolated, split for measurement with a Yokogawa OSA, and directed through a circulator (which provided additional isolation) to a linear dispersive medium (fiber Bragg grating) to create amplitude mode-locked optical pulses, then passed through a LiNbO_3_ modulator driven by a 24 GHz sine wave generator (Anritsu MG3695B) to down-convert the comb beating frequency, and subsequently received by a linear photodetector (Thorlabs PDA8GS). The RF linewidth of the detected spectrum was acquired over 60 s, measured with an electrical RF spectrum analyzer (Tektronix RSA5103B), and fit to a Voight profile to extract the mode beating linewidth (full-width half max) of all the comb laser lines (Fig. 7).

### Comb laser RIN measurements

**Figure 8:**
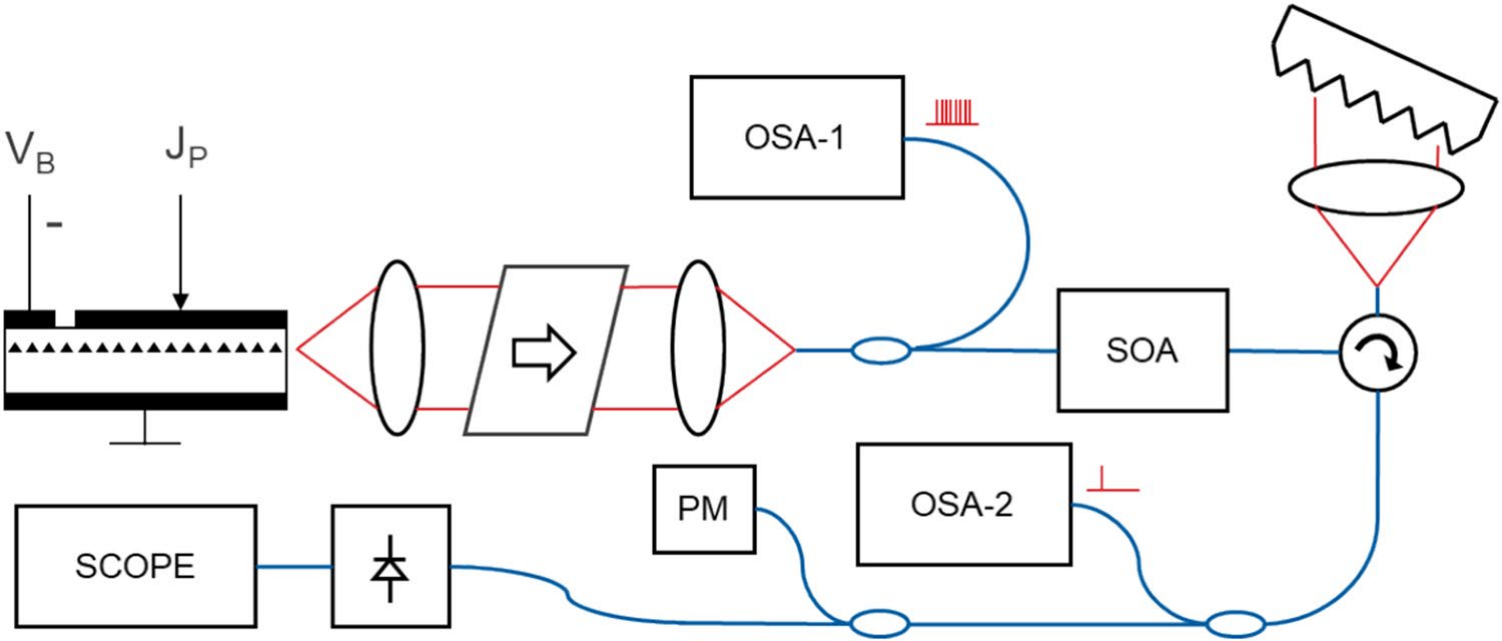
Setup for RIN measurement showing clockwise from top left the FP comb laser with drive and absorber bias sections, a free space isolator and fiber coupler (in a laser butterfly package), a 10/90 splitter with an OSA and a semiconductor optical amplifier (SOA), a circulator with a tunable free-space grating filter, a 10/90 splitter with a second OSA, a 10/90 splitter with a power monitor and a high-speed photodetectorwith a chirped fiber bragg grating reflector, a high-speed MZI (24 GHz) driven by a signal oscillator (OSC) for down-conversion of the comb beat frequency, a 10/90 splitter with a power monitor (PM) and variable optical attenuator (VOA) followed by a high-speed photodetector and an electrical spectrum analyzer (ESA). PM fibers used (blue lines).

Relative intensity noise measurements were taken for the entire comb by feeding the comb laser output to a DC-coupled linear 10 GHz photodetector (Thorlabs PDA8GS) connected to a 50 GHz sampling oscilloscope (Infiniium DCA-J 86100C). The ratio of the variance of the signal to its mean identifies the RIN averaged from DC-10 GHz under the assumption of normal distribution of samples in the recorded oscilloscope trace. The RIN of individual comb lines were measured by filtering them with automated narrowband filter tunable across 120 nm with a wavelength resolution of 20 pm, a full-width-half-maximum bandwidth of 0.1 nm, and an out-of-band suppression in excess of 45 dB (Fig. 8).

### Comb laser linewidth measurements and analysis

The comb mode linewidth was measured using a well-known self-heterodyne method^36^. The measured spectrum in our case includes components of noise associated with the driver, photodetector, and measurement apparatus. The 3 dB linewidth measurement is dominated by 1/f noise at frequencies close to the fundamental^37^ and dominated by white noise envelopes further away (e.g. 20 dB linewidth). The measured spectrum is hence a convolution of the approximately Gaussian spectrum arising from the 1/f noise with the Lorentzian spectrum arising from the white frequency noise, following the well-known Voigt profile. The convolution of the 1/f noise sources can lead to an overestimation of the intrinsic or natural Lorentzian (white-noise-limited) linewidth of the laser from the measured spectrum. However, it is possible to numerically extract the natural Lorentzian linewidth from the measured RF spectrum by carefully fitting the Voight function across several decades of power and subsequently extracting the linewidth of the Lorentzian part of the Voight profile^38^.

**Figure 9:**
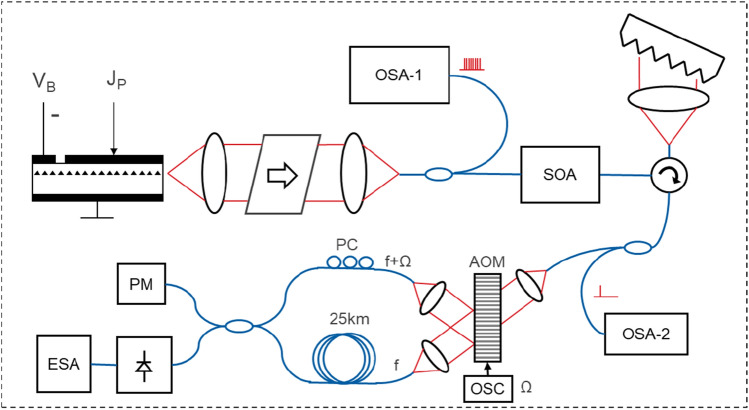
Setup for linewidth measurement of an individual comb mode showing clockwise from top left the FP comb laser with drive and absorber bias sections, a free space isolator and fiber coupler (in a laser butterfly package), a 10/90 splitter with an OSA and a semiconductor optical amplifier (SOA), a circulator with a tunable filter, a 10/90 splitter with a second OSA and an acousto-optic modulator (AOM) driven by an electrical signal oscillator (OSC). The modulated output of the AOM was coupled to a polarization controller (PC) and interfered with the unmodulated output of the AOM delayed through 25 km of single mode fiber. A 2 × 2 coupler was used to equally split the output between a PM and a high-speed photodetector and ESA.

Measurements were taken by isolating individual comb lines using a narrowband filter with a FWHM of 0.1 nm and using a self-heterodyning method to determine the linewidth of the individual comb lines. To significantly reduce supply noise, the laser drive current was supplied with a 12 V battery-based source for the duration of the measurement. Reverse bias on the absorber section was also applied with a 1.5 V battery. The individual laser mode was propagated through free-space acousto-optic modulator (80 MHz center frequency), which split in two arms, the modulated one and unmodulated one, the second one was delayed via a fiber loop of 25 km (SMF-28) and interfered with a modulated arm. The beat signal from photodetector was measured with an electrical RF spectrum analyzer. By fitting a Voight function to the measured data over 4 decades of power (Fig. 6) the natural Lorentzian-linewidth of the laser was numerically computed from the fit. Two modes were measured and analyzed using this method, resulting in 1/f (Gaussian) noise limited linewidths of 340 kHz and 367 kHz as well as intrinsic (white-noise limited) Lorentzian linewidths of 140 kHz and 170 kHz respectively (Fig. 9).

## Data Availability

The datasets generated and analysed during the current study are available from the corresponding authors on reasonable request.
